# Quality assessment, inclusive community development, and collective learning: An institutional perspective from Germany

**DOI:** 10.3389/fresc.2022.890822

**Published:** 2022-08-26

**Authors:** Albrecht Rohrmann, Johannes Schaedler

**Affiliations:** Center for Planning and Evaluation of Social Services (ZPE), University Siegen, Siegen, Germany

**Keywords:** intellectual disability services in Germany, quality assurance, inclusion, path, drivers for innovation, quality dialogue, local planning, collective learning

## Abstract

This article reviews the German discourse on quality of life, quality assurance, and outcome measurement in services for persons with intellectual disabilities. Following institutional assumptions of path dependencies in organizational development, it is argued that concepts such as quality assurance must be understood in the context of the national support system development. For the Federal Republic of Germany, it can be noted that previous approaches to quality assurance of services based on measurement and evaluation tools have not been the drivers of innovation for inclusion. The driving forces behind reforms in the field of disability originated from the three angles of the social service structure (people with disabilities, statutory welfare agencies, and service providers). Policies of key actors were not part of a consistent reform strategy. However, the main elements of the inclusive philosophies of the disability rights movement became hegemonial and led to national legislation that prioritizes person-centered support arrangements in inclusive settings. With regard to governance arrangements in Germany and the idiosyncrasies of local disability fields, it is suggested that there should be a conceptualization of quality assurance in a multilevel approach as “local quality dialogues for collective learning.”

## Introduction

Quality development measures are often expected to provide strong impulses for improving the life conditions of people with disabilities, especially for people with intellectual disabilities. In this context, the focus of attention is exclusively directed to the quality of support practices within single-service organizations. The UN Convention on the Rights of Persons with Disabilities (CRPD), particularly the requirements of Article 19, has provided an effective international impulse for many countries toward political and professional measures to promote independent living. Nevertheless, its implementation in each national context is based on the respective developmental paths of social welfare systems and differ from country to country. This ground must be understood in the international discourse on concept development for improving the quality of life of people with disabilities.

The development in the Federal Republic of Germany is characterized by the fact that there is a legal entitlement to social benefits for people with disabilities. This is regulated at the federal level and services are financed by governmental agencies. But, in the field of service provision, non-governmental associations, such as those of churches, dominate. They are called intermediary organizations because they combine the characteristics of state organizations, of enterprises, and of organizations of the voluntary sector. They are granted a high degree of autonomy in the design of assistance and traditionally have a strong influence on how society deals with the target group they support. They often claim to represent the rights of people with disabilities in an advocatory manner.

Regardless of the federal legal basis, there are very large regional differences in the infrastructure of support services for people with disabilities. This is related, for example, to the tradition of large institutions, the regionally varying activities of lobby groups for inclusion, and the policy of municipalities and other regional stakeholders.

The example of the development in the Federal Republic of Germany shows that an isolated discussion of quality in service organizations in general cannot make a substantial contribution to improving the quality of life for people with disabilities. Such a perspective would focus on only on the interaction between professionals and beneficiaries and would ignore the context of this relationship. If, on the other hand, looking at the drivers of innovation to comply with the requirements of the UN CRPD, albeit hesitantly and sometimes contradictorily, it becomes apparent that the structures of the support system become crucial for change. The structures refer to the financing of services, the management of services in individual cases, the rights of the beneficiaries, and the integration of support services into the regional community. Reviewing the development of the support system in Germany, therefore, is to contribute to the understanding of the structural dimension in the quality debate.

## The governance context of the German service system for persons with intellectual disabilities

In order to understand how the discourse on “quality of life” and “quality of services” and “outcome measurement” has been received in the field of services for people with intellectual disabilities in Germany, it is necessary to look at the governance system in this field and its developmental paths until today. When speaking of “fields,” we relate to neo-institutional approaches in organizational analysis (1–3). An organizational field can be defined as “those organizations that, in the aggregate, constitute a recognized area of institutional life: key suppliers, resource and product consumers, regulatory agencies, and other organizations that produce similar services or products” (4, p. 64). On this basis, it can be assumed that single disability services are interconnected with other disability service organizations in their region and thus form a field. Such fields share conceptual assumptions, knowledge, have specific forms of interaction, power structures, and are aware of a common purpose. With regard to the developmental paths of organizations and their fields, it seems plausible that history matters, i.e., “initial choices preclude future options, including those that would have been more effective in the long run (…) Altering institutional rules always involves high switching costs, thus a host of political, financial and cognitive considerations mitigate against making such changes” (5). Recognizing path dependency can explain why paradigmatic changes of given institutional practices in disability services are so hard to realize in practice.

Therefore, in the following, key aspects of the development of services for people with intellectual disabilities in Germany will be summarized.

Intellectual disability services that Germany developed in the 19th century in the framework of religious organizations that rather early formed umbrella organizations: the protestant actors established the “Innere Mission” (1848), which later became the “Diakonie,” and on the catholic side in 1898, the “Caritas” was founded as a joint association of catholic initiatives (for the deeper political context, see ref. 3, pp. 21–94). At the turn of the 19th to the 20th century, there were approximately 80 religiously run “imbecile institutions” in Germany, each one often caring for more than 1,000 people (6). The running of the large facilities was mostly supported by order sister and brothers, i.e., voluntary and unpaid workforce of unmarried women and men who acted with a strong religious orientation focusing on physical care, work, and religious education.^1^ In the early 1920s after WW I, the political system in Germany changed from monarchy to republic and the religious welfare organizations were joined with other welfare organizations from the labor movement (*Arbeiterwohlfahrt*, Workers' Welfare Association) *Deutsches Rotes Kreuz*, Red Cross, the *Paritätische Wohlfahrtsverband*, Independent Welfare Association, and the *Zentralwohlfahrtsstelle der Juden*, Jewish Welfare Organization)^2^ to form a powerful overall third sector umbrella organization “*Liga der Freie Wohlfahrtspflege*” (“League of non-statutory welfare”).

Since then, the relation between the state and the non-statutory welfare organizations is defined by the so-called “principle of subsidiarity,” which in simple terms means that whatever the individual, family, group, or organizational body can do for themselves is not to be left to the responsibility of the government. This principle became a structural element of later German social legislation with far-reaching consequences. Until today, it obligates the German government agencies to leave the provision of all kinds of social services to the non-statutory, free voluntary welfare organizations, while the state remained responsible for meeting relevant costs. All service (8) providers at the local level were expected to be a member of one of the six welfare associations listed above. As a result, the provision structures in social work in general are shaped by non-governmental associations that run most^3^ of the various services, whereas the main role of governments is that of the funder. Moreover, the non-statutory welfare sector still has a legally guaranteed conceptual autonomy on how to provide services as long as this remains within the framework of the legal prescriptions of the Social Code Book (SGB).

The field of intellectual disabilities is structured just like this, whereby the religious organizations nationwide are still the biggest players in disability service provision, followed by parents' organizations and others. Which provider association is dominant in a certain region differs according to given local developmental paths with origins in local social milieus and religious traditions. Despite marketization policies that had started in the mid-1990s, in the field of intellectual disability, there are almost no private service providers with a for-profit orientation (8).

For the context of this article, it is important to note that governance structures and financing of services for persons with intellectual disabilities in Germany are shaped in a triangular relationship between the individual persons as the beneficiaries, the state, and the service providers. The national government regulates eligibility conditions of beneficiaries for the different service areas through the national Social Code Book IX that have to be implemented by the sixteen federal states and by local governments. In order to receive the services they are legally entitled for, people with intellectual disabilities have to go through an application procedure that is based on the assessment of their individual needs. This application procedure leads to a legal claim of a beneficiary against the government to pay for eligible services provided by non-governmental welfare organizations. Healthcare and long-term care needs of people with intellectual disabilities are covered by social insurance schemes and are part of the general social protection system. Most other services for persons with intellectual disabilities that offer support, e.g., in day-to-day living, employment, or leisure time are part of the social assistance system under the “Integration Act” and therefore means-tested; in reality, private funding or out-of-pocket payment plays only a marginal role.

When in the 1970s the debate on deinstitutionalization in mental health services received high public attention, also the large care institutions for persons with intellectual disabilities came under growing critique. With some delay, the Scandinavian principle of normalization as conceptualized by Bank–Mikkelsen, Grunewald, or Nirje (see all their contributions in 9) was intensively discussed in the 1980s. However, in practice, it affected the German service system for people with intellectual disabilities only in a “moderate” way, i.e., primarily as a professional guideline, thus widely ignoring both its dimension of citizen rights and its sociopolitical ambition to improve the living conditions of persons with disabilities. Roughly speaking, the appropriateness of segregating facilities such as special kindergartens, special schools, residential homes, or sheltered workshops was not generally questioned. Instead, contradictions between concepts and institutional practice in the field were often summoned under unprecise normalization wordings, i.e., that services should allow persons with disabilities “obtain an existence *as close to* the normal as possible” (Bank–Mikkelsen). Still, especially parents' associations all over Western Germany, felt supported by the principle of normalization and engaged successfully in establishing group homes (with mostly 24 places in three groups) as an alternative to large institutions. As a result, gradually more of such residential homes for persons with intellectual disabilities were added to the traditional institutional system, but often based on the assumption that people with severe and profound disabilities were better off in large institutions.

While as in other Western countries, also in Germany, the disability rights movement became stronger and conceptual critique on large institutions and the segregating support system for people with disabilities also became more influential. However, the high autonomy of voluntary welfare organizations from governmental influence was still unanimously defended by most relevant actors from across the voluntary welfare sector. For principal reasons, it was requested that the state should remain in the role as a funder for social welfare and governments should not interfere in conceptual issues such as service models. Moreover, approaches of governments to make providers to report on the quality of their services were branded as an illegitimate element in the legal system (10). Even though when pressure on policymakers, e.g., from disabled people's organizations campaigning against segregating institutions and for new inclusive service models had mounted in the early 1990s, many government actors tried to avoid conflicts with the non-governmental service sector. Also because of corporatist structures reflecting the strong influence of the voluntary welfare sector in social politics, there was not much public political interest in substantial reforms for systematic dismantling of large institutions and building a community service system.

Following neoliberal ideas from the US and UK in the 1990s, both government actors and welfare organizations also in the disability sector came under the influence of new public management philosophies. While culture and routines in services became more “managerial” in nature, government actors on different political levels attempted with new legislation to release themselves from the role of the mere funder in order to make the system more cost-effective. According to the slogan “value for money,” concrete steps were undertaken by policymakers to implement market elements such as purchasing and commissioning in social service provision and to implement a financing system based on contracts with service providers (11). As part of the contract conditions of the new funding system for the disability services of 1994, service providers were expected to provide high-quality services and to document these by establishing internal quality assurance schemes.

It was a widespread assumption of bureaucrats in the welfare administration and among service providers that such instruments for quality assurance would have the potential to function as motors for modernizing the institutionalized system of services for persons with intellectual disabilities. In search of orientation on how to conceptualize the quality assurance in disability services, two main routes were taken:

The direction of one route led to an international discourse on quality of life (QoL) and quality of services (QoS) that had reached the German intellectual disability field *via* publications of the International League of Parents Organizations (ILSMH).^4^ Approaches to measure QoL were rather regarded as a contribution to the value base of service providers and were conducted with a strong ceremonial interest but not for systematic development of the service organizations. The issue “quality of services” was received in the tradition of Wolfensberger's instruments PASS or PASSING (12), which claimed to measure “how normalizing are current human services” (13). With some adaptions, comprehensive instruments for large institutions^5^ were developed by German provider organizations, often with a strong focus on staffing issues (14). These instruments were mostly regarded as part of a strategy to improve the position of service providers in the funding negotiations with the government (11).

An exception was the so-called “LEWO- instrument,” which became widely used as a method to develop the quality of services in group homes (15). It followed the idea of guided self-evaluation, providing professional standards for good support and management of services that were to be matched in a multistakeholder evaluation team with given practices in order to come to internal recommendations for developing the quality of life of users.

The other direction, in which actors look for conceptual orientation on how to assure the quality of services for persons with disabilities, was led by quality management systems. These approaches were inspired either from quality assurance schemes in the industrial sector or from corresponding models in other fields of human service delivery, mainly healthcare in hospitals. In the context of upcoming managerialism in services for people with disabilities, approaches such as “Total Quality Management” (TQM) were used to install quality management systems in many service organizations. These QM systems were based on a “quality-handbook” in which key processes of service provision were described as a compulsory orientation for staff. Services were expected to perform with better quality and higher cost-efficiency when establishing such QM systems with regular audits and certification according to industrial norms. In the same context, Donabedian's model of assessing of service quality (16) obtained a leading function also in the field of intellectual disability services. The model was originally developed for rating and ranking the quality of US hospitals and discriminates between “structure,” “process,” and “outcomes”:

*Structure* refers to the resources used in the provision of care, and to more stable arrangements under which care is produced; *process* refers to the activities that constitute care; and the *outcomes* are the consequences to health that were referred to in the proceeding section. (16, p. 6)

Donabedian's dimensions are still of use when it comes to describing and analyzing services for people with intellectual disabilities in Germany. Also, in some disability services, QM systems are still existing. But in practice, both approaches have lost relevance and generally speaking, often led to rather technical approaches. This was due to the fact that the implementation of quality assurance schemes could not effectively support the claims of service providers for better staffing in negotiations with governments. Also, the QM approaches failed to be consistent with regard to quality standards for structures. While focusing on processes in services, they tended to ignore the crucial meaning of the institutional setting itself for people with intellectual disabilities being at risk of institutional discrimination. Thus, quality assurance concepts as such could not contribute to substantially transforming the widely specialized residential care system for persons with intellectual disabilities into service models that comply with the inclusive paradigm.

This does not mean that during this period no progress toward inclusive services models was achieved. Indeed, in the early 2000s, new service models based on individual support arrangements for persons with intellectual disabilities living alone or with a mate in their own apartments were initiated by innovative service providers all over the country. This process contributed to the development of a parallel system of institution-based care and community care. It followed the logic of an additive pattern of change, i.e., more and more inclusive services were established, while residential homes and institutions widely remained as they were, which seems to be typical for reforms in corporatist governance arrangements such as in Germany.^6^

Reformers again were rather optimistic when policymakers introduced concepts like “self-determination” and “equal participation” of people with disabilities in the national Rehabilitation Law 2001. Also, new funding options for services for persons with disabilities such as “personal budgets” were established in order to give beneficiaries more choice and strengthen their position as service users. However, the expected effect, that people with disabilities in great numbers would vote with their feet, i.e., against care in larger institutions and go for self-directed care arrangements, has not been realized. This can be attributed to bureaucratic hurdles to utilization and restraint on the part of provider organizations, but also raises the question of whether market control can replace the systematic planning and development of services (18, p. 136 f.).

Progress for more inclusive service models was achieved through local initiatives from the disability rights movement who took the impulses from the UN CRPD after its German ratification 2009 and campaigned against discrimination and for new inclusive service models also for persons with intellectual disabilities. The Federal Participation Act (Bundesteilhabegesetz, BTHG) that came into force in 2017 can be seen as another political effort to reform the services and assistance provided for persons with disabilities. The Participation Act has been constructed “in the light of UN-CRPD” and aims at putting the beneficiary at the center of service provision. It intends to overcome the parallel system of institutional and community care by prioritizing the development of inclusive services across the lifespan, e.g., for family support, for inclusive education, for supported living, or for supported employment. At the same time, the Participation Act again wants to increase the possibilities of government actors to steer service delivery processes and strengthen the position of governments in the triangular system of service provision.

Summarizing the documented reform efforts, it can be stated for Germany that the institutional cornerstones of the triangular governance structure of services for people with intellectual disabilities have remained stable over time. The inherent institutional persistence of the corporatist setting has made modernization policies difficult but has also protected the sector from neoliberal austerity policies that could have led to major cuts in the funding of services. Still, existing large institutions find themselves under continuing critique and are trying to compensate their massive legitimation deficits with various organizational strategies. However, in the last few decades, inclusive services offering support in inclusive education, supported living, supported employment, various forms of personal assistance for independent living, etc., have been established all over the country serving people with all kinds and degrees of impairments.

As has been shown, approaches for quality assurance in services based on measurement and assessment instruments have not been the motors of this overall development toward inclusion. But then, what have been its drivers and what relevance could fall on quality development approaches?

## Drivers of innovations

As has been explained, the governance structure in the field of services for people with intellectual disabilities in Germany is characterized by a remarkable persistence against institutional change toward inclusive models. However, in addition to institutional care in large and small residential facilities over time, new service models have been implemented across the country that allow people with intellectual disabilities to live independently and be included in their communities. The driving forces behind this development have been very different and are not part of a consistent reform strategy. They rather result from activities in all the three angles of the social service triangle (beneficiaries, statutory welfare agencies, and service providers) with very different motivations and policies. We think the following “drivers” can be identified, which are only loosely coupled with quality.

### Disability rights movement and user control

Despite many setbacks, the disability rights movement has succeeded in gaining public support for a non-discriminative policy. That has been institutionalized step by step, e.g., in a ban on discrimination against people with disabilities in the German constitution and individual entitlements for inclusive services and legal requirements for accessible environments. The disability rights movement has also had a strong impact on the support system for people with disabilities (19). This was achieved on the basis of new rights-based assumptions and philosophies on the purpose of support services with consequences for assessment and measurement of their outcomes:

As has been outlined above, over years, beneficiaries have been demanding more influence in the development and design of services, so that they allow more user control and higher flexibility with maximum self-determination. In many services that were founded in the last two decades by innovative service providers (20, p. 7), the importance of people's own home became the focus and was also developed for people with intellectual disabilities. By separating the rental relationship of a client with disability from the support relationship, the right for privacy was to be realized and maximum user control ensured. This puts structural criteria for the organization of services in the foreground, while professional considerations on quality of services become second in importance. In this perspective, professional concepts for measuring quality of life even with general indicators tend to be viewed critically because they might call the individual autonomy of persons with disabilities into question. It is believed that in weighing user control against the limitations of organizational practices, services should respect people's rights for participation and support individual lifestyles even when considered as undesirable or even risky by experts.

According to the new Participation Act, all services should enable independent living. This also sets a new orientation for the discussion on quality standards. Positive outcomes of support are not primarily to be measured by the quality of the work processes in facilities but by the facilitation of participation and independent living of persons with intellectual disabilities. Thus, the structural features of support services such as flexibility, local availability, and avoidance of dependency become more important. Moreover, when reflecting about standards, the safeguarding of user control and self-determination also become most relevant.

The conceptual assumptions of inclusive services, however, are based on individual rights but do not agree with mere market philosophies. The latter do not adequately understand the fact that services cannot be established only when an individual need is articulated, but in a welfare state, arrangements must be available as part of a public social infrastructure. This shifts the focus of the quality discussion away from the individual service organization to the development of a local service system with different services and support offers.

### Government's steering by contract management and individual service planning

With all inherent contradictions, it can be stated that government welfare agencies have successfully claimed more influence and control on the provision of services in the field of intellectual disability in Germany. This development can be seen in the context of the economization of the provision of social services, which oriented policymaking toward independent living that constrained the institutional power of large care institutions and their political networks. It also offered incentives for institutional change for traditional service providers and support for new social entrepreneurs with innovative concepts.

Government actors have chosen two different approaches for this: (a) contracts with service providers and (b) individual planning procedures with beneficiaries.

Ad (a): In 1994, the national government changed the funding basis of social services supporting people with disabilities. Earlier, service providers could bill the government welfare agency for their costs after providing services, whereas since then, they must enter into a contract for a future period. The contract also contains an agreement on quality assurance measures. However, no inspection requirements were placed on the measures, and control effects remained limited. Nevertheless, it can be assumed that the use of instruments for contracting and quality assurance has contributed to increasing the transparency of the service provision.

Ad (b): Governments have started to exercise more control on the assessment of individual needs as part of the application procedure of beneficiaries for services. The aim is to ensure that person-centered assistance is granted rather than standardized care packages, e.g., a place in a group home. For this purpose, welfare agencies have developed instruments for individual service planning and such “planning procedure” (§ 117 SGB IX) has to be carried out as a compulsory part of each application procedure. For the planning procedure, an impressive list of quality criteria was specified by the legislator: accordingly, the procedure must be transparent, interagency, interdisciplinary, consensus-oriented, individual, lifeworld-oriented, social space–oriented, and oriented to individual goals.

Only the beneficiaries and statutory welfare agencies are to be involved in this process of needs assessment and service planning, while service providers are not to participate in order to avoid conflicts of interests. However, the beneficiaries can consult a person they trust, so service providers might have access to the process this way. In the case of children and adolescents, the public youth welfare agency is tob be involvedand in the case of long-term care needs the long-term insurance agency is to be involved. At the center of the procedure is a systematic needs assessment, which refers to all nine domains of life of the International Classification of Functioning, Disability and Health (ICF). On this basis, needs are identified and a plan is drawn up that is binding on service providers. In their service agreement, the services commit to aligning their support with the support plan. The implementation of the plan is monitored and updated *via* the agreed objectives.

At the time of writing, the new individual planning process has not yet been fully implemented. Many statutory welfare agencies lack qualified staff to carry out this challenging task. In practice, therefore, individual planning is often re-delegated to service providers. Also, as before the reform, service contracts with service providers are still based on standardized service packages, which often are not related to individual goals. Notwithstanding these difficulties, the implementation of the new procedures for service agreement and individual support planning has already had a significant impact on the quality discussion. Procedural questions of correctly assessing needs, setting appropriate goals, and negotiating appropriate services have become main challenges.

With regard to measuring the effectiveness of support, the individual support plan and its objectives to improve participation become the key document. The monitoring of individual objectives for equal participation implies a conceptual departure from measurable indicators of quality of life. Following assumptions of what is called the “the capability approach” (21), the purpose of reflection, given service practices, is about enabling participation in different areas of social life. It should be noted, however, that the possibilities of equal participation cannot be achieved through quality support of one service alone. Participation is possible only if the structures of the housing market, the education, and the socioeconomic system offer opportunities for equal participation at the local level.

### Development of services and isomorph processes

The traditional providers of services have also taken up the reform impulses from professional debates and diversified their service structures. Almost all have now added counseling and supported living services to their portfolio. They have modernized their profile from charity organizations to social enterprises. How come? One explanation can be found in the fact that the providers of service providing organizations depend on resources from their external environment. In order to ensure that resources are continuously provided on a safe basis, service organizations are interested in meeting the expectations of other relevant actors. Such legitimation, of course, must come from government funding agencies but also from other stakeholders of the field and from the general public. As Richard Scott from the perspective of organizational sociology put it: “In institutional environments organizations are rewarded for establishing correct structures and not for the quantity and quality of their outputs” (2, p. 167). Therefore, service organizations must make sure that they are “acting on collectively valued purposes in a proper an adequate manner” (5, p. 185) which makes them sensitive for changing expectations in their environment concerning how modern services for people with intellectual disabilities should operate. Certainly, traditional care organizations will be interested in stability, in maintaining their internal power structure, and in avoiding transition cost and therefore use their autonomy to avoid change. However, when service organizations become aware that other service organizations in their field offering inclusive models gain positive attention and public recognition for reasons of legitimation, they will tend to go with their practices isomorphic in the same direction as the “successful others.”

To sum up the argument, the more the inclusive paradigm in providing services for people with intellectual disabilities became hegemonial, the more even very conservative service providers were forced to change their service models. This process supported the diffusion of innovative service models, and today, it can be observed that some forms of segregating institutional facilities, i.e., large institutions or group homes, are being retained. Service providers also offer apartments for small groups of people with disabilities, for couples or individuals, where the tenancy is linked to the provider. This development must be viewed critically with regard to the requirements of the UN CRPD, especially with regard to Article 19 and its interpretation by the Committee for disabled persons of the United Nations (22). However, an altogether developmental dynamic toward decentralized flexible support services can be seen.

With regard to the quality discussion, it is significant that again the dimension of structural criteria (16) is gaining importance. Smaller units with rules that are conceptually oriented to private housing are supposed produce higher quality of services. With the shift from focusing on structures and not on processes of client–staff interaction, an improvement in the quality of life of the users is expected.

These developments, which focus in different ways on the position of users, government agencies, and service providers, are by no means free of tension to one another. What they have in common is that they shift the focus away from services as self-containing units to service systems and accessible environments. This seems to require a redefinition of what exactly should be the subject of quality assessment. When services are seen in the perspective of the UN CPRD as a part of “appropriate measures” for people with intellectual disabilities to enable participation and inclusion on an equal basis with others, the focus of assessing and developing outcomes should be widened from the single service organization to local service fields and community infrastructure. Approaches to this will be outlined in the following.

## Measuring and assessing quality in a community development perspective

It can be assumed that the purpose of quality development is to improve the living conditions of people with intellectual disabilities and to further develop the day-to-day routines of service provision in a given local region. Thus, the main function of measuring the outcome of given practices is to allow a reflection on their strengths and weaknesses in order to identify steps for improvement. This needs suitable methodologies for assessment of services and of the living conditions of their clients, but it also has implications on which actors should be involved in what formats. With regard to the highly structured governance arrangements and the idiosyncrasies of local disability fields, we suggest a conceptualization of such processes as “local quality dialogues for collective learning.”

In the following, three levels will be distinguished at which a new impulse for quality development can start: the individual level of enabling self-determination and independent living, the level of quality management in services, and the level of local networks and infrastructure for enabling participation. We feel that on each level such quality dialogues should be based on quality standards and indicators that allow assessment, and this assessment should be done in multistakeholder settings in which people with disabilities have a strong voice. Furthermore, quality standards and assessment procedures on the different levels should be closely coupled with mechanisms to translate recommendations into practice. Moreover, altogether, they should follow a consistent policy of raising the living conditions of people with disabilities, a policy that is coordinated by local governments.

### The individual level

The quality of services for people with intellectual disabilities is determined in particular by the extent to which they allow independent living. In the German welfare state system, beneficiaries must apply for legal entitlements in order to gain access to services. While earlier the application procedure was about finding a place in an institution, the procedure now starts with assessing the will, wishes, and needs of a person. This leads to an individual support plan that forms the basis for both public financing of the service arrangement and the measurement and monitoring of its effectiveness with regard to the given objectives.

Such individual assessment procedures are very complex endeavors and need high professional expertise. Particularly when connected with diagnoses, they can cause shaming and stigmatization of people with disabilities seeking assistance. Therefore, quality criteria are needed to define how the procedures for needs assessment can be carried out in a non-discriminatory manner. Following the UN CRPD, disability can be understood as a result of interaction between people with impairments and barriers in the environment. The assessment of support needs can, therefore, no longer be based on the characteristics of a person with impairments only. It also must also consider the context factors in the environment of the person that hinder or promote active participation in all domains of day-to-day life. Therefore, quality standards and indicators are needed to relate to such procedural requirements. They should also create a basis for the joint evaluation of the given support arrangement. Furthermore, it seems necessary for improving the quality of the service provision to establish an institutional link between individual planning procedures for a person with disabilities and the development of an inclusive social environment in the given community.

### The level of services

From what has been said, we argue that quality development at the level of service organizations should be based on professional standards that comply with the human rights model of disability and the prescriptions of the UN CRPD. These standards should be discussed and negotiated in a participatory manner with all relevant actors to promote ownership on compliance with the standards in use. This should also include a reflection on needs for further development of the residential service organizations themselves. For the development of suitable standards and criteria it is possible to use existing concepts and approaches (see contributions in ref. 23).

The formation of user interest groups has widely become a standard in housing services that is increasingly safeguarded by corresponding legal requirements. When establishing quality circles or evaluation teams for quality development in service organizations, the participation of users should become a standard. The assessment of practices should not be limited to internal processes within the service organization. Of course, strategic decision-making, e.g., about the future profile of the organization, will remain the preserve of management and supervisory bodies. However, participation practices and even peer evaluation by users can contribute to reproducing a segregating framework of institutions if they do not reach the level of service development.

The UN's Committee for Disabled people recommends “a strategy and a concrete plan of action for deinstitutionalization” (22, p. 11) for the development of service organizations in the direction given by Article 19 UN CRPD. Such action plans for continuous inclusive development of services are to be developed in a participatory way. This can become a part of structural quality management of services. This would shift the focus of quality assurance to overcoming segregating practices in residential facilities for persons with disabilities. Experiences show that quality measurement activities focusing only on single services probably soon reach their limits. The main quality criteria are, when single services understand themselves as part of a regional network of support services and locate their activities in the context of a coordinated effort to develop inclusive communities. In the US context, some decades ago, similar ideas were discussed under the term “communitization” (24).

### The level of local support networks and infrastructure

A self-determined life is realized in social relationships. The accessibility and usability of the local social environment is of particular importance when people have to cope with disabilities and major social dependencies. Their locality with its very concrete conditions is where participation in everyday life, in education, in leisure time, or in employment is realized. This is also true even when decisions about, e.g., education systems or inclusive labor markets are made at other levels. Since some time, it can be observed that the use of digital media and assistive technology is becoming increasingly important for social participation. Gaps in digital participation lead to new social divisions with high risks for people with intellectual disabilities (25).

In Germany, there is a widely developed legislation regulating support services for people with disabilities in the form of Social Code Book IX. However, these contain only weak specifications for the planning of service systems at the local level. While in many municipalities and districts local action plans exist to develop accessibility and inclusive infrastructure, this is still not well linked to the field of disability services. However, both the accessibility and usability of the physical infrastructure and the accessibility of digital technology are critical for inclusion and full participation of people with disabilities. Quality standards for a local infrastructure that enables people with disabilities to live self-determined lives should relate to the following aspects:

• Appropriate housing, educational and employment opportunities, and recreational activities to meet diverse needs without discrimination.
• The accessibility of public space for all.
• Self-advocacy and support groups to represent interests in the community.
• Counseling services, including peer counseling, to assist in organizing an independent living in all areas of life.
• Decentral organized services for support in everyday life.

With these standards, the focus of quality discourse changes. It is not only the quality of a single service that is relevant for opportunities to live a self-determined life included in society but the structures and living conditions in the community. This brings local governments in an important position as they represent the political level closest to the citizen and are responsible for providing quality services and inclusive infrastructure in their territory. Systematic planning processes at the community level that are coordinated by local governments become a central quality requirement for the implementation of a rights-based approach for disability services. This also refers to the United Nations' Sustainable Development Goals where Goal 11 calls to “Make cities and human settlements inclusive, safe, resilient and sustainable” (https://sdgs.un.org/goals/goal11).

There is already knowledge and practical experience on the methodologies that can be used to assess and develop the quality of services and local infrastructure in the context of inclusive community planning (26, 27).

## Conclusion

As we have shown, the governance structure in the field of services for people with intellectual disabilities in Germany is characterized by a remarkable persistence against institutional change toward inclusive models. However, in addition to institutional care in large residential facilities, over time, new service models have been successfully implemented across the country that allow people with intellectual disabilities to live independently and be included in their communities. Approaches for quality assurance in services based on measurement and assessment instruments have not been the motors of this development toward inclusion. Rather, the driving forces behind this development stem from activities in all the three angles of the social service triangle (beneficiaries, statutory welfare agencies, and service providers), but they were not part of a consistent reform strategy. This does not mean that approaches for quality assessment are generally regarded as ineffective, but we suggest a widening of their focus from single services to local service fields and inclusive infrastructure. With regard to the highly structured governance arrangements in Germany and the idiosyncrasies of local disability fields, we also suggest a conceptualization of quality assessment as “local quality dialogues for collective learning.” These local dialogues should be initiated on three levels: (a) the individual level of enabling self-determination and independent living, (b) the level of quality management in services, and (c) the level of local networks and infrastructure for enabling participation. When developing standards and indicators for assessing the quality of a given local situation, we argue that with reference to Donabedian's model, the dimension of “structures” is crucial if person-centered support arrangements are to be realized.

## Data Availability

The original contributions presented in the study are included in the article/Supplementary Material, Further inquiries can be directed to the corresponding author.

## Footnotes

^1^During the Nazi regime, they failed to counter the eugenic movement and the euthanasia crimes (7).

^2^After having been dissolved like the other welfare umbrellas by the Nazi regime, the Jewish Welfare Association had already been reestablished in 1951, and today, it has a nationwide network again (see https://zwst.org/de).

^3^The exemption is the field of long-time care services for the elderly, where private-for-profit providers count for ca. 50% of services.

^4^Later, its name was changed to *Inclusion International*.

^5^For an overview see (17) SYLQUE (System zur Entwicklung von Lebensqualität in Einrichtungen für Behinderte) or GBM (Gestaltung der Betreuung von Menschen mit Behinderungen) see (17).

^6^On the other hand, as being persistent against institutional change, the corporatist settings did not allow the implementation of neoliberal austerity policies that have led to major cuts in funding of services in countries with more marketized social sectors.
